# Comparison of infarct volume and behavioral deficit in Wistar Kyoto and spontaneously hypertensive rat after transient occlusion of the middle cerebral artery

**DOI:** 10.1186/2193-1801-2-414

**Published:** 2013-08-28

**Authors:** Jorge Garcia, Jon Dang, Pardes Habib, Cordian Beyer, Markus Kipp

**Affiliations:** Institute of Neuroanatomy, Faculty of Medicine, RWTH Aachen University, Wendlingweg 2, 52074 Aachen, Germany

**Keywords:** tMCAO, SHR, Wistar, Stroke, Behavior, WKY, Wistar

## Abstract

Rodent models of focal cerebral ischemia are important tools in experimental stroke research. Such models have proven instrumental for the understanding of injury mechanisms in cerebral stroke and helped to identify potential new therapeutic options. A plethora of neuroprotective substances have been shown to be effective in preclinical stroke research but failed to prove effectiveness in subsequent clinical trials. Interestingly, preclinical studies have shown that neuroprotective agents are selectively effective in different rat strains. The underlying mechanisms for this discrepancy are so far unknown, but differences in initial stroke volume with concomitant neuroinflammatory processes in the expanding stroke area might be relevant.

In the current project, we compared the stroke volume and behavioral outcome between Wistar Kyoto (WKY) and spontaneously hypertensive rats (SHR), subjected to transient middle cerebral artery occlusion (tMCAO) for 1 h, followed by 23 h reperfusion. We further analyzed the expression of well-known pro-inflammatory mediators in the cortical peri-infarct area region using a TTC-based isolation approach.

Initial reduction of local cerebral blood flow was comparable between both strains. Mean infarct volume and the extent of tMCAO-provoked functional deficits did not differ between WKY and SHR rats. Furthermore, the induction of pro-inflammatory mediators, among CCL3 and CCL5, in the isolated ischemic peri-infarct area region was equal in both rat strains.

We were able to demonstrate that stroke outcome is comparable 23 h after transient MCAO in WKY and SHR rats. Future studies have to show whether this observation confirms in the long-term, and which factors contribute to differences observed with respect to therapeutic responsiveness.

## Background

Cerebral stroke, also called cerebrovascular accident, is the third leading cause of death and causes among the most prevalent neurological diseases severe physical disability in adulthood worldwide. Major risk factors for embolic stroke are hypertension, age, nicotine abuse, dyslipidemia, and *Diabetes mellitus* (Grau et al. 2001). Recombinant tissue plasminogen activator (tPA) is the only approved medication in acute ischemic stroke (Group TNIoNDaSr-PSS 1995). However, less than 20% of all stroke patients receive tPA which is mainly owed to the narrow treatment time window of therapeutic success (Weimar et al. 2006).

Rodent models of focal cerebral ischemia are essential tools in experimental stroke research. They have contributed significantly to our understanding of cell death mechanisms in stroke and helped to identify potential new therapeutic strategies. A plethora of neuroprotective substances have been shown to be effective in preclinical stroke research using animal models, but failed to exert beneficial effects in subsequent clinical trials (Braeuninger and Kleinschnitz 2009). Several factors may be responsible for the failure of translation from bench to bedside. There are significant physiological, neuroanatomical, and metabolic differences between humans and small rodents, which are the most common used experimental animals in preclinical stroke research. Furthermore, preclinical stroke studies are often restricted to juvenile male animals, although it is evident that age significantly affects stroke outcome (Davis et al. 1994,1995; Duverger and MacKenzie 1988). Metabolic disturbances such as hypertension or hypertriglyceridemia might also impact on stroke outcome and might influence the effectiveness of any therapeutic intervention. Interestingly, direct side-by-side comparison of neuroprotective compounds revealed striking strain differences in their potential to influence stroke outcome. Porritt et al. (2010) for example demonstrated that angiotensin-converting enzyme (ACE)-inhibition reduces the infarct area in normotensive but not hypertensive rats. In another study, 17beta-estradiol increased stroke damage in normotensive rats with no significant effect in stroke-prone rats (Carswell et al. 2004). Other variables which make an interpretation of different studies difficult include sex, age, and other comorbidities (Braeuninger and Kleinschnitz 2009).

Preclinical anti-stroke drug development was often conducted with the Wistar Kyoto (WKY) rat strain, but stroke patients often suffer from metabolic disorders and/or hypertension. Therefore, rats with comparable metabolic stress might better mimic the human situation. Spontaneously hypertensive rats (SHR) share many metabolic problems with essential hypertension in humans and appear to be suited for a comparative study. SHR rats have a genetic background of WKY and suffer from early hypertension, hyperinsulinemia, hypertriglyceridemia, and hypercholesteremia (Iritani et al. 1977; Swislocki and Tsuzuki 1993). Consequently, the SHR strain recently is more frequently used in stroke and cardiovascular research (Gow et al. 2011; Wang et al. 2012).

In the current project we compared the stroke volume and behavioral outcome between the two different rat strains, i.e. WKY and SHR. Both were subjected to tMCAO for 1 h, followed by a reperfusion of 23 h before sacrification. Besides determining the infarct volume and behavioral deficits, we analyzed the expression of well-known pro-inflammatory mediators in the cortical peri-infarct area region using TTC-based RNA isolation (Dang et al. 2011; Kramer et al. 2010).

We were able to demonstrate that stroke outcome is comparable 23 h after transient MCAO in WKY and SHR rats.

## Results

### CBF course and infarct volume in WKY and SHR rats

**Figure 1:**
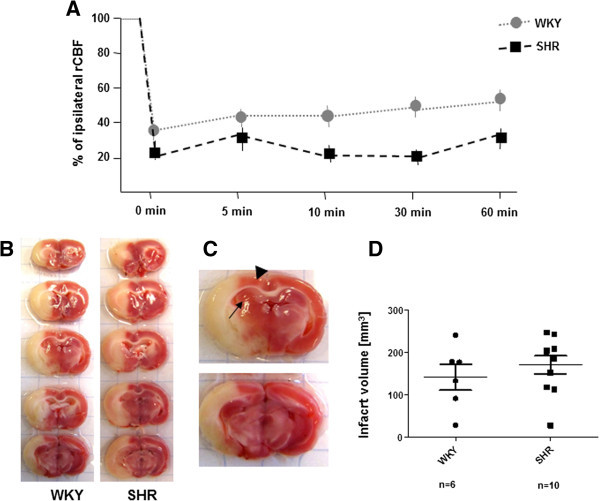
**rCBF and cortical infarct volume. (A)** Representative Laser-Doppler measurement of relative cerebral blood (rCBF) flow over the ipsilateral middle cerebral artery territory before and during tMCAO (1 h). Reduction of rCBF is given as percentage of baseline values. **(B)** TTC-stained 2 mm coronal brain slices show the white infarct area 24 h after tMCAO. Red color indicates intact tissue. **(C)** Arrowhead indicates the cingulated cortex, arrow the hippocampus. Quantitative data of the measurement of lesion volumes are given in **(D)**. Note that lesion volumes are comparable in WKY and SHR rat strains. Data represent means ± SEM. For further abbreviations see text.

To analyze the influence of disturbed cardio-vascular and metabolic parameters for stroke outcome, tMCAO was performed in WKY and SHR rats following the same standardized protocol. Laser-Doppler monitoring revealed that in all rats included in the study, regional CBF values were reduced by >60% compared to pre-ischemic values after induction of tMCAO and remained stable during the entire occlusion period. As shown in Figure 1A, no significant differences were observed in the regional CBF between both strains. Mean values dropped ~80% in SHR and ~68% in WKY compared to baseline values. Furthermore, focal cerebral perfusion remained at levels approx. 50% of the baseline during the entire occlusion in both strains. Please note that SHR show a slightly larger, but not significant drop of CBF than WKY, nevertheless all animals remained at CBF levels we previously determined as sufficiently low for ischemic damage after experimental tMCAO.

In the next step, we investigated the infarct volume in TTC-stained coronal brain sections 23 h after reperfusion. TTC staining reliably delineated the infarct region allowing to discriminate between the hypoxic-damaged pale- compared to the surrounding red non-injured area. Figure 1B depicts the morphological appearance of the infarcted brain areas of two series of representative brain slices corresponding to WKY and SHR rats subjected to tMCAO. The lesion is confined in both strains to the ipsilateral striatal region and the anterior and posterior cerebral cortical vascular MCA territories as indicated by the unstained pale area. Medial orientated cortical areas, corresponding to the cingulated cortex (arrowhead in Figure 1C) as well as the ipsilateral hippocampus formation (arrow in Figure 1C) were not affected in both rat strains. Subsequent quantification of the cortical infarct volume, corrected for edema formation, revealed no significant differences between both strains, although a slightly larger infarct volume is seen in SHR, which might correlate with the larger drop of CBF in SHR. The infarct volume was 141.85 ± 30.51 mm^3^ for WKY and 168.73 ± 21.54 mm^3^ for SHR.

### Behavioral deficits in WKY and SHR rats

**Figure 2:**
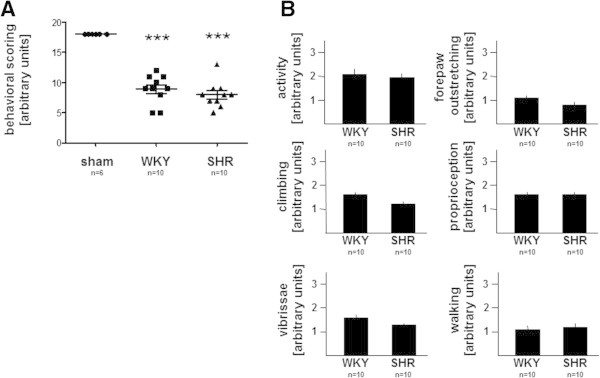
**Behavioral testing. (A/B)** Results of behavioral testing of WKY and SHR rats 24 h after initiation of tMCAO. A total of six sub-tests were performed resulting in a maximum of 18 scores in sham-operated animals (no tMCAO). Note that behavioral outcome is comparable in both rat strains. Data represent means ± SEM. For further abbreviations see text. ***p< 0.001 sham compared to tMCAO.

In addition to the parenchymal infarct damage, we evaluated stroke-induced functional impairments in both strains after tMCAO. Besides spontaneous activity, different motor, coordination, and sensory aspects were tested (see methods section). As shown in Figures 2A/B, tMCAO significantly decreased the maximum scoring from 18 points (sham-operated animals without tMCAO) to ~9 in both rat strains. Not just that the overall behavioral scoring declined to a comparable extent in WKY (8.9) and SHR (8.0) animals (see Figure 2A), but scoring of all subcategories was comparable as well, as demonstrated in Figure 2B. Taken together, infarct volume and tMCAO-related behavioral deficits are comparable in WKY and SHR rat strains.

### Expression of pro-inflammatory mediators in the peri-infarct area of WKY and SHR rats

**Figure 3:**
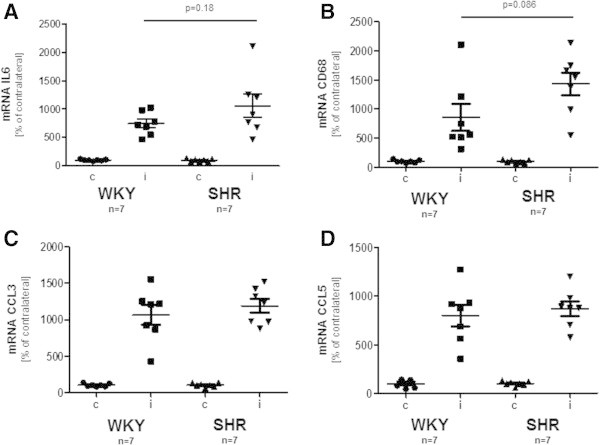
**Gene expression in the peri-infarct area.** Gene expression studies in tissue obtained from the cerebral cortex peri-infarct area after tMCAO. **(A)** interleukin 6 expression, **(B)** CD68 expression, **(C)** chemokine (C-C motif) ligand 3 expression, **(D)** chemokine (C-C motif) ligand 5 expression. Data represent means ± SEM. For further abbreviations see text.

Under pathological and anatomical aspects, the peri-infarct area is defined as the area surrounding an ischemic event resulting from ischemic, thrombotic or embolic stroke. A widely accepted definition for the peri-infarct area is “ischemic tissue potentially destined for infarction but not yet irreversibly injured and the target of acute therapies” (Fisher and Bastan 2012). It has been shown that differences exist in the architecture and composition of the peri-infarct area between WKY and hypertensive rat strains (Reid et al. 2012). This prompted us to analyze in greater detail the expression levels of well-defined pro-inflammatory mediators during stroke, namely interleukin-6 (IL-6), CD68, CCL3 (MIP1α), and CCL5 (RANTES) by means of rt-PCR. As demonstrated in Figure 3, the expression of all factors was significantly induced and up-regulated in the peri-infarct area 23 h post tMCAO. IL-6 and CD68 show a slightly higher expression in SHR, but in accordance with the data obtained for behavioral recovery and infarct spread, no significant differences in expression levels were found between WKY and SHR rats (p=0.18 for IL-6 and p=0.086 for CD68). Control experiments revealed that no significant regulation of these factors is evident in the contra-lateral hemisphere compared to sham-operated animals (data not shown).

## Discussion

Despite extensive animal research concerning the pathophysiology of focal ischemic brain injury, no effective translational treatment protocol emerged for stroke therapy. A large number of neuroprotective agents have been studied in the past such as free radical scavengers, excitatory amino acid antagonists, hypothermia, barbiturates, calcium channel blockers or growth factors (Green 2008). Many of these agents appeared protective or curative in preclinical studies with small-animal models of ischemia (rats, mice, or gerbils), but unfortunately none of these have proven conclusively to be effective in humans (Green 2008; Savitz and Fisher 2007). An important critical aspect is the choice of the appropriate animal model keeping in mind that none would exactly mimic human stroke. Many experimental studies used young WKY rats for preclinical anti-stroke drug development (Dang et al. 2011; Melani et al. 2009; Ortega et al. 2012). However, the “typical” stroke patient is elderly and suffers from several co-morbidities such as hypertension, dyslipidemia *et cetera*. In this study, we have compared the infarct volume, behavioral deficit, and the expression of inflammatory markers in the peri-infarct area between SHR and WKY animals during the initial infarct period until 24 h after ischemia onset. Such a direct comparison between both rat strains is important for our understanding of stroke pathology and why neuroprotective strategies might be effective in one species but not in another.

Although, we have to emphasize that a tendency towards a higher decline in rCBF and increased expression of CD68 and IL6 was observed in SHR rats, our analysis revealed that all tested parameters were similar in both strains. According to the literature, certain rodent strains are more sensitive to MCAO and produce more sizable infarction volumes, whereas other strains produce only a small infarction with MCAO (Takaba et al. 2004). For example Hom et al. (2007) reported that SHR rats show increased infarct volume following MCAO compared with WKY rats. However, in contrast to the current study protocol, they performed permanent MCAO and infarct volumes were analyzed after a relatively short period (4 h post occlusion) which significantly might impact on the post-stroke outcome.

There is no doubt that significant differences exist between different rat strains that are commonly used during preclinical stroke studies. SHR is a classical animal model for studying hypertension, whereas stroke-prone spontaneously hypertensive rat (SHRSP) are more frequently used as spontaneous experimental model for human stroke. Both SHR and SHRSP are hypertensive animals, although SHRSP is more susceptible to stroke as compared with SHR. In one study, gene expression profiling in the brains of SHR and SHRSP was comparatively analyzed by gene chips. Of the 76 differentially expressed genes under physiological conditions, 41 genes were up-regulated, and 35 genes were down-regulated in SHRSP as compared with SHR. One factor that showed reduced expression in SHRSP rats was a riboflavin kinase which was subsequently identified as a new potential risk factor for stroke (Zou et al. 2012). In another study, it has been demonstrated that E-selectin levels in brain tissue and serum are significantly higher in SHRSP compared to SHR rats after a high salt diet. The authors were subsequently able to demonstrate that E-selectin deficiency improves neurological behavior and reduces the infarction area after MCAO (Ma et al. 2012). Porritt et al. (2010) were able to show that angiotensin-converting enzyme (ACE)-inhibition reduces infarction in normotensive but not in hypertensive rats. WKY rats in the cited study differed from SHR with respect to their marked cortical ACE activity that is highly sensitive to ACE inhibition. Interestingly, the beneficial effect of ACE inhibition on the infarct volume in normotensive rats does not correlate with changes in blood pressure. In another study, Carswell et al. (2004) presented data that MCAO-induced brain damage is reduced in SHRSP but not in normotensive WKY rats during proestrus. Thus, a systematic comparison of different rat strains which differ with respect to stroke development appears as a useful approach to identify candidate genes related to stroke pathophysiology.

Besides behavioral outcome and stroke volume, we analyzed the expression of well described pro-inflammatory mediators in the cortical peri-infarct area region using a novel TTC-based isolation approach (Dang et al. 2011; Kramer et al. 2010). We focused on such factors, i.e. IL-6, CD68, CCL3 and CCL5, which have already been described by us and others occurring in the ischemic peri-infarct area and being relevant for tMCAO-induced brain damage and post-stroke therapy (Benakis et al. 2012; Bleilevens et al. 2013; Dang et al. 2011; Ginsberg 1997; Kriz and Lalancette-Hebert 2009). We were able to show that the magnitude and signature of gene expression up-regulation is comparable within the peri-infarct area of both rat strains, although we would like to emphasize that there is a tendency to higher expression profiles of CD68 and IL-6 in SHR rats. Nevertheless, the similar outcome of stroke volume, behavioral restoration, and important pro-inflammatory markers suggest that there is no significant difference with respect to short-term recovery between both rat strains. There are several important issues, we would like to underline. First, we have no data concerning the long-term outcome in both strains up to 1–2 weeks. It is well known that the infarct area has not yet fully matured 24 hours post tMCAO (Neumann-Haefelin et al. 2000) and it thus might be that differences in stroke outcome become evident during later time points. The same applies for functional deficits which might become worse over time, as well. Second, animal numbers used in this study are small and it might well be that minor differences in stroke volume might be significant if higher animal numbers were used. Third, in our study we restricted our analysis to the cortex and did not include subcortical regions such as the striatum. In a recently published study we were able to demonstrate that female sex steroids are able to protect cortical but not sub-cortical areas after tMCAO in rats (Dang et al. 2011), suggesting that cortical areas are more sensitive for neuroprotective trial as subcortical areas are. Furthermore, it has been shown that functional test scores were not distinguishable between sham-operated animals and those with exclusive caudoputaminal infarcts (Wegener et al. 2005) underpinning the importance of cortical areas for post-stroke evaluation.

## Conclusion

Nevertheless, our data convincingly demonstrate that stroke outcome using the tMCAO model provides comparable values in WKY and SHR rats 23 h post reperfusion.

## Methods

### Animals

10–12 weeks old male WKY and 13–15 weeks old male SHR (i.e. comparable weight) were purchased from Charles River (Germany) and maintained according to the rules of “Care of Animal Subjects” (North Rhine-Westphalia, Germany) in a pathogen free and climate-controlled environment with free access to water and food *ad libitum*. The Review Board for the Care of Animal Subjects of the district government reviewed and approved the research and animal procedures. Behavioral testing and infarct volume quantifications were performed in a blinded manner. The experimenter was not aware of the genetic background of the animal and animal from both strains were randomly selected to avoid any external influence on experimental outcome.

### tMCAO and monitoring of cerebral blood flow

Most preclinical studies use the transient intraluminal thread method for transient middle cerebral artery occlusion (tMCAO) (Bleilevens et al. 2013; Dang et al. 2011; Ulbrich et al. 2012). This model was originally developed in rats, and has subsequently been modified (Belayev et al. 1996; Chu et al. 2008) and adapted to mice. In this model a monofilament is advanced via the common or external carotid artery into the internal carotid artery towards the division of the anterior and middle cerebral arteries and thus occludes the middle cerebral artery vascular territory. The filament may be removed allowing for reperfusion (transient MCAO, tMCAO) or may be left in place permanently (permanent MCAO). The advantages of this technique are that it models focal infarction in a large vascular territory without craniotomy (Braeuninger and Kleinschnitz 2009).

In our experiments rats were subjected to general anesthesia with 5% isoflurane (Abbott, Ludwigshafen, Germany) for 2–3 min and maintained for further surgical steps at 2–2.5%. Body temperature was monitored and maintained at 37°C ± 0.5°C using a rectal control system coupled with a heating plate and infrared illumination. Laser-Doppler measurement (Moor Instruments, VMS-LDF2, Axminster, UK) of the local cerebral blood flow (CBF) at the ipsi- and contralateral side was used to assure a successful occlusion of the middle cerebral artery (MCA). For Laser-Doppler monitoring, the skull was exposed and the sensors were positioned at ~1-2 mm posterior and ~4-5mm lateral of the bregma (based on the highest blood perfusion units (BPU)). tMCAO was performed as previously described (Dang et al. 2011; Kramer et al. 2010; Ulbrich et al. 2012) with minor modifications which were required due to minor anatomical differences between both rat strains. Following a small midline neck skin incision, the left common carotid artery (CCA), internal carotid artery (ICA) and external carotid artery (ECA) were exposed and the vagal nerve was carefully separated from the CCA. Subsequently, the ECA was mobilized, small arteries, including the minor occipital artery were cauterized and finally cut. After detaching the *musculus omohyoideus* from its tendinous origin, the ICA was mobilized until the origin of the *arteria pterygopalatina* from the ICA was clearly visible. Subsequently, the exposed *arteria pterygopalatina* was ligated and mobilized by a filament to allow its manipulation. The ECA was transiently clipped. Subsequently, the proximal CCA was permanently ligated, a small incision in the CCA performed, and a commercially available catheter (0,014'' Asahi PTCA Guide Wire Soft, Abbott Vascular, Abbott Park, IL, USA) introduced into the lumen of the CCA. The catheter was carefully pushed cranially under visual control until the tip of the catheter passed the origin of the *arteria pterygopalatina*. The head was then bend aside and the catheter was gently pushed further forward until a significant drop (>60% compared to baseline values) of the local CBF occurred. After successful occlusion of the MCA, the clipping of the ECA was removed. After 1 h, the catheter was removed and the CCA proximally and distally to the incision ligated. Finally, the ligation of the *arteria pterygopalatina* was released.

Sham-operated animals were subjected to the same surgical steps, the catheter was not introduced into the CCA.

### Behavioral testing

A set of motor and sensory behavioral tests which are commonly used to evaluate outcome in this model were used to evaluate post-ischemic neurological deficits 23 h after tMCAO (Garcia et al. 1995). The following 6 tests were performed:

For analyzing spontaneous activity, the rat was placed in an unfamiliar environment (35 cm × 55 cm sized cage) and behavior was observed for 1 min. (3 = moving around, exploring cage, and approaching at least 3 sides of the cage; 2 = slightly affected, moving but hesitating to explore all the cage, still approaching at least one side of the cage; 1 = obviously affected, slightly moving around but not reaching any of the cage’s walls; 0 = not moving).

The outstretching of the forepaws was evaluated by fixating the tail and comparing the outstretching of the forelimbs (3 = symmetrical outstretching of both forepaws; 2 = less outstretching of the right side but still moving both forelimbs; 1 = slight movement of the right side; 0 = no movement of right forepaw).

To test the climbing ability, animals were placed on the wall of a wire cage (3 = climbs and uses all four limbs and grabbing wire tightly; 2 = climbing but with evident loss of ability and power on right side; 1 = not climbing, not holding to the wire, or circling instead of climbing).

Walking coordination and walking activity (3 = walking straight ahead; 2 = not moving; 1 = walking to the right side; 0 = circling right).

The sensory test for impaired proprioception was performed by touching both sides of the rats with a blunt stick and comparing the reactions to the stimulus of either side of the body (3 = turning the head and moving to side of the stimulus, symmetrical reaction on both sides; 2 = reacting but slowly to touch on right side; 1 = no response to stimulus on right side).

Reaction to stimulus of the vibrissae was used to complete the sensory testing (3 = turning head to side of the stimulus symmetrically; 2 = reacting slowly to touch on right side; 1 = no reaction to touch on right side).

After adding all individual test scores, a total maximum score of 18 and a minimum total score of 3 were achievable.

### TTC staining and quantification of infarct volume

23 h post-tMCAO, rats were sacrificed and transcardially perfused with physiological NaCl. The brains were quickly removed and placed in a cutting matrix (Alto Brain Matrix stainless steel 1 mm rat coronal 300-600 g; Harvard Apparatus, Holliston, MA, USA). Five coronal slices with an individual thickness of 2 mm were cut and incubated for 20 min in a 2% (w/v) TTC (2,3,5-triphenyl-tetrazoliumchloride) solution prepared with physiological NaCl. TTC has been shown to be a reliable marker for metabolic active tissue because it is chemically reduced to red TPF (1,3,5-triphenylformazan) in the presence of dehydrogenases (Altman 1976; Kramer et al. 2010). We used TTC to delineate the ischemic core region from the peri-infarct area and to assess the infarct volume. After the TTC staining, slices were arranged in a frontal-occipital orientation, and digital images were taken using a Canon Digital IXUS 9015 Camera (Canon, Tokyo, Japan). The freely available software ImageJ 1.44 p (NIH, Bethesda, MD, USA) was used for the morphometric evaluation of the infarct volume. The following equation was used for infarct volume measurement and edema correction as published previously (Dang et al. 2011; Kramer et al. 2010):

### Genexpression analysis in the cortical peri-infarct area region

In an ischaemic territory, irreversible damage progresses over time from the center of the most severe flow reduction to the periphery with less disturbed perfusion. This centrifugal progression of irreversible tissue damage is characterized by a complex cascade of interconnected electrophysiological, molecular, metabolic and perfusion disturbances. This region is called ‘peri-infarct area’ and was in the focus of our gene expression studies. Gene expression studies were solely performed with tissues corresponding to the peri-infarct area region defined in TTC-stained sections as published previously (Kipp et al. 2008; Dang et al. 2011; Kramer et al. 2010; Clarner et al. 2011). In brief, the cortical peri-infarct area was manually dissected using a stereomicroscopic approach, dissolved in lysis buffer (NucleoSpin_ RNA/Protein kit, Macherey-Nagel, Germany), and homogenized with 1.4 mm diameter ceramic beads (Precellys 24, Peqlab, Germany) at 5,000 rpm for 2 × 15 s. RNA was subsequently isolated from this peri-infarct area tissue following a recently published protocol (Dang et al. 2011). Purity was controlled using 260:280 OD ratios (Nano- Drop 1000, Peqlab, Germany). Gene expression was measured using the RT-qPCR technology (BioRad, Germany), 2× SensiMix Plus SYBR & Fluorescein (Quantace, Germany) and a standardized protocol as described previously (Acs et al. 2009; Braun et al. 2009; Baertling et al. 2010). Relative quantification was performed using the ΔCt method which results in ratios between target genes and the housekeeping reference gene hypoxanthine guanine phosphoribosyltransferase (HPRT). Melting curves and gel electrophoresis of the PCR products were routinely performed to determine the specificity of the PCR reaction (data not shown). Primer sequences and individual annealing temperatures are given in Table 1.

**Table 1:** **Primer sequences and annealing temperatures (AT)**

Gen	Sequenz	AT [°C]
HPRT	s 5*'* - GGTCCATTCCTATGACTGTAGATTTT - 3*'*	60
	as 5*'* - CAATCAAGACGTTCTTTCCAGTT - 3*'*	
CD 68	s 5*'* - AATGTGTCCTTCCCACAAGC - 3*'*	60
	as 5*'* - GGCAGCAAGAGAGATTGGTC - 3*'*	
IL 6	s 5*'* - ACAGTGCATCATCGCTGTTC - 3*'*	60
	as 5*'* - CCGGAGAGGAGACTTCACAG - 3*'*	
CCL 2	s 5*'* - CCAGAAACCAGCCAACTCTC - 3*'*	58
	as 5*'* - CCGACTCATTGGGATCATCT - 3*'*	
CCL 5	s 5*'* - TGCCCACGTGAAGGAGTATTTTTA - 3*'*	60
	as 5*'* - TGGCGGTTCCTTCGAGTGACAA - 3*'*	

### Statistical analysis

6 WKY and 10 SHR were included in infarct volume measurements, 10 WKY and 10 SHR were included in behavioral testing and 8 WKY and 8 SHR were included in gene expression analyses. Statistics for infarct volume and gene expression studies were performed using absolute data. Intergroup differences were tested by one-way ANOVA analysis and independent t-test using GraphPad Prism 5 (GraphPad Software Inc.). For behavioral analyses, we performed the non-parametric Mann–Whitney test. *P*-values are indicated as * = *p* ≤ 0.05, ** = *p* ≤ 0.01, *** = *p* ≤ 0.001.

## Authors’ information

Beyer C and Kipp M are senior authors.
